# Association of maternal snuff use and smoking with Sudden Infant Death Syndrome: a national register study

**DOI:** 10.1038/s41390-022-02463-4

**Published:** 2023-02-09

**Authors:** Anna Gunnerbeck, Cecilia Lundholm, Samuel Rhedin, Ayoub Mitha, Ruoqing Chen, Brian M. D’Onofrio, Catarina Almqvist

**Affiliations:** 1 Department of Medical Epidemiology and Biostatistics, Karolinska Institutet, Solna, Sweden; 2 Neuropediatric Unit, Department of Women’s and Children’s Health, Karolinska Institutet, Solna, Sweden; 3 Sach’s Children and Youth Hospital, Stockholm, Sweden; 4 Clinical Epidemiology Division, Department of Medicine Solna, Karolinska Institutet, Solna, Sweden; 5 Université de Paris, Epidemiology and Statistics Research Center/CRESS, INSERM (U1153 - Obstetrical, Perinatal and Pediatric Epidemiology Research Team (EPOPé)), INRA, Hôpital Tenon, Bâtiment Recherche, Paris, France; 6 School of Public Health (Shenzhen), Sun Yat-sen University, Guangzhou, China; 7 Institute of Environmental Medicine, Karolinska Institutet, Solna, Sweden; 8 Department of Psychological and Brain Sciences, Indiana University, Bloomington, IN USA; 9 Pediatric Allergy and Pulmonology Unit, Astrid Lindgren Children’s Hospital, Karolinska University Hospital, Solna, Sweden

## Abstract

**Background:**

The aim was to study whether non-combustible nicotine (Swedish snuff) use in pregnancy is associated with elevated risk of post neonatal mortality, Sudden Infant Death Syndrome (SIDS), and Sudden Unexpected Infant Death (SUID) and to study how cessation before the antenatal booking influenced these risks.

**Methods:**

This was a population-based register study of all infants with information on tobacco exposure in early pregnancy born in Sweden 1999–2019, *n* = 2,061,514. Self-reported tobacco use in early pregnancy was categorized as nonuse, snuff use, and moderate and heavy smoking. Multiple logistic regression models were used to estimate crude and adjusted odds ratios (aORs) with 95% confidence intervals (CIs).

**Results:**

Maternal snuff use was associated with increased risks of post neonatal mortality, SIDS, and SUID. The risks of snuff use and moderate smoking were of similar magnitude. Heavy smoking was associated with the highest risks. Cessation of smoking and snuff use before the antenatal booking was associated with lower risks of SIDS and SUID compared to that of continuous usage.

**Conclusions:**

Maternal snuff use was associated with increased risks of post neonatal mortality, SIDS, and SUID. Nicotine is the common substance in cigarette smoke and snuff. These findings support the hypothesis that nicotine contributes to an elevated risk of SIDS.

**Impact:**

- Maternal snuff use and smoking in early pregnancy were associated with increased risks of post neonatal mortality, SIDS, and SUID.
- Cessation of smoking and snuff use before the first antenatal visit was associated with reduced risks of SIDS and SUID.
- The common substance in cigarette smoke and snuff is nicotine. Our findings suggest that nicotine contributes to an elevated risk of SIDS and SUID.
- The implication of our findings is that all forms of nicotine should be avoided in pregnancy.

## Introduction

Maternal smoking is one of the most important preventable risk factors for infant morbidity and mortality and associated with increased risk of Sudden Infant Death Syndrome (SIDS).^1,2^ SIDS is a cause assigned to infant deaths that cannot be explained after a thorough case investigation including autopsy, a scene investigation and a review of clinical history. Due to more advanced forensic techniques in recent years, there has been a diagnostic shift in many countries with increased reporting of accidental suffocation and strangulation in bed and a subsequent decline of reported cases of SIDS.^3^ Thus, the term Sudden Unexpected Infant Death (SUID) is frequently used, including SIDS, unspecified death, and accidental suffocation and/or strangulation.^4^ Although the risk of SIDS is multifactorial, maternal smoking, following sleeping position, remains the strongest modifiable risk factor for SIDS in developed countries.^1,4^ SIDS is suggested to be related to a disturbed cardiorespiratory control and a blunted response to hypoxia with impaired arousal.^1^ Animal studies of prenatal nicotine exposure have shown similar disturbances in the cardiorespiratory control as seen in infants exposed to smoking in pregnancy, which suggests that nicotine is involved in the mechanisms behind smoking-related risks of SIDS.^5^

With increasing knowledge about the hazardous effects of smoking on women’s and children’s health, smoking in pregnancy has declined in Sweden from 13% to <5% between 1999 and 2019.^6^ Non-combustible nicotine products are regarded less harmful than cigarette smoking, and as a consequence the use of smokeless tobacco and electronic nicotine delivery systems (ENDS) has increased during the past decades.^7^ Swedish snuff is an oral moist powder and contains equally high levels of nicotine as cigarettes, but no combustion products and only low levels of tobacco-specific nitrosamines.^8^ Snuff use among women has increased substantially the past decades and daily snuff use in Sweden is approximately 7% of women in reproductive age.

However, among women aged 16–29 years snuff use has tripled from 3% in 2018 to 9% in 2021.^9^ Approximately 1.8% of Swedish women used snuff in pregnancy in 2021.^9^ Whereas sufficient knowledge about nicotine exposure from electronic cigarettes in pregnancy still are lacking,^10,11^ maternal snuff use has been associated with increased risk of stillbirth, preterm birth, neonatal apnea, and disturbed heart rate variability.^12–15^ However, in contrast to smoking, snuff use has not been associated with increased risk of early neonatal mortality.^14^ Whether nicotine, in the form of Swedish snuff, increases the risk of post neonatal mortality and SIDS has to our knowledge not previously been studied.

The aim of this study was to investigate the associations of snuff use and smoking in early pregnancy with post neonatal mortality and SIDS. Because of the increased use of the terminology SUID in many countries, we chose to study both SIDS and SUID. Further, we wanted to study how cessation of snuff use or smoking in very early pregnancy, before the first antenatal visit, influenced these associations.

## Methods

### Study population

**Fig. 1:**
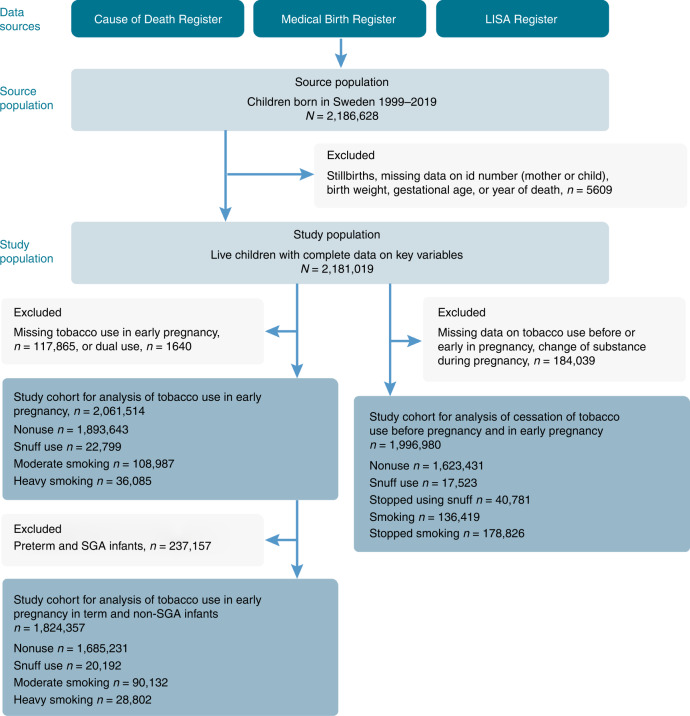
Schematic presentation of the study population. Flow chart of the source population, exclusions, the final study population as well as categorization of exposures.

Based on the Swedish Medical Birth Register (MBR), we included all live-born infants, born between 1999 and 2019, with information on gestational age, birth weight, and mothers’ individual identification number, *n* = 2,181,021 births. Infants who died during the first year of life, but with invalid date of death, were excluded, *n* = 2. The eligible study population was 2,181,019. We excluded infants of mothers who had missing information on tobacco use in early pregnancy (*n* = 117,865). Dual users (users of both cigarettes and snuff) were too few (*n* = 1640) to be analyzed separately and were also excluded. A total of *n* = 2,061,514 births were included in the analyses with tobacco use in early pregnancy as exposure, Fig. 1.

The MBR is a national register containing information on the demography, maternal reproductive history, pregnancy, delivery, and neonatal period for 98% of all births in Sweden. By means of the unique personal identification number, the MBR can be linked to other data sources such as the Cause of Death Register and the Longitudinal integrated database for health insurance and labor market studies (LISA) and the Register of Total Population. The Cause of Death Register includes all deaths of Swedish citizens with annual updates, while LISA includes information on education for 98% of Swedish residents.^16^ Since 1997, diagnoses in the MBR and the Cause of Death Register are classified according to the 10th Revision of the WHO International Classification of Diseases (ICD-10).

### Snuff use and smoking in pregnancy

Self-reported data on maternal smoking have been registered in the MBR since 1982 and maternal snuff use since 1999. Information on present tobacco use and use 3 months before pregnancy was collected at the antenatal booking, which generally occurs between gestational weeks 8 and 12. Maternal tobacco use was categorized as nonuser (no tobacco use), moderate smoker (1–9 cigarettes /day), heavy smoker (≥10 cigarettes/day), and snuff user (yes/no). Information on the number of pouches of snuff used daily is lacking in the MBR, which only allowed us to categorize women as user or nonuser.

To investigate whether cessation of tobacco use in very early pregnancy, before the antenatal booking, influenced the risk of post neonatal mortality, SIDS, and SUID, we combined information on tobacco use 3 months before pregnancy with use in early pregnancy. Exact quit date of tobacco use was not possible to obtain. Mothers who neither used tobacco before nor in early pregnancy were categorized as nonusers. Snuff users 3 months before pregnancy and in early pregnancy were categorized as persistent snuff users. Smokers 3 months before pregnancy and in early pregnancy were categorized as persistent smokers. Mothers who used snuff or smoked before pregnancy but not at the antenatal booking were categorized as quitters of snuff or smoking, respectively. We excluded infants of mothers lacking information on tobacco use before and/or in early pregnancy, dual users, and mothers who changed substance of tobacco or who started to use tobacco during pregnancy (*n* = 184,039). A total of 1,996,980 live births were included when studying cessation of tobacco use before the antenatal booking (Fig. 1).

### Post neonatal death, SIDS, and SUID

Post neonatal mortality was defined as death between 28 and 364 days. SUID is a term used to describe any sudden and unexpected death occurring before 1 year of age. In Sweden, unexpected infant death is thoroughly investigated with anamnesis, medical history, examination, and laboratory testing. However, in contrast to some others countries crime scene investigations are not routinely performed in Sweden.^17^ Autopsy of the infant is routinely performed by the forensic pathologist and the diagnosis reported to the Cause of Death Register.^18^ After case investigation, if SUID cannot be explained otherwise, it is categorized as death before 365 days from birth and including SIDS (R95), unspecified causes (R99), or strangulation/suffocation in bed (W75). SIDS was categorized as death before 365 days and ICD-10 R95.

### Covariates

Covariates were chosen based on their association with both tobacco use in pregnancy and neonatal death in previous research.^2^ Preterm or small for gestational age (SGA) birth is highly associated with both tobacco use, post neonatal mortality, and SIDS and potentially on the causal pathway between the exposure and the outcome. Thus, preterm and SGA births were considered possible mediators for the associations of tobacco use and risks of post neonatal mortality, SIDS, and SUID. SGA was defined as birth weight below the tenth percentile for gestational age according to population-based birth weight standards. Preterm birth was defined as birth before 37 gestational weeks. We retrieved information on maternal age, parity, co-habitant with father-to-be, the child’s gestational age and birth weight from the MBR, and information on mother’s level of education from LISA. Mother’s country of birth was obtained from the Register of Total Population. The categorization of covariates are shown in Table 1.

**Table 1:** Maternal and birth characteristics for tobacco use in early pregnancy, *N* = 2,061,154.

	No use	Snuff use		Moderate smoking		Heavy smoking	
	*N* = 1,893,643, *n* (%)	*N* = 22,799, *n* (%)	*p* value^a^	*N* = 108,987, *n* (%)	*p* value^a^	*N* = 36,085, *n* (%)	*p* value^a^
*Maternal characteristics*							
Maternal age (years)			*p* < 0.0001		*p* < 0.0001		*p* < 0.0001
<20	21,430 (1.1)	349 (1.5)		6027 (5.5)		1255 (3.5)	
20–24	216,101 (11.4)	3491 (15.3)		28,104 (25.8)		6966 (19.3)	
25–29	584,145 (30.9)	7136 (31.3)		33,269 (30.5)		10,133 (28.1)	
29–34	670,004 (35.4)	7078 (31.1)		25,094 (23.0)		9715 (28.9)	
≥35	401,963 (21.2)	4745 (20.8)		16,493 (15.1)		8016 (22.2)	
Parity			*p* < 0.0001		*p* < 0.0001		*p* < 0.0001
1	823,078 (43.4)	9250 (40.6)		48,605 (44.6)		11,024 (30.6)	
2–3	961,569 (50.8)	11,710 (51.4)		50,313 (46.2)		18,146 (50.3)	
≥4	108,996 (5.8)	1839 (8.1)		10,069 (9.2)		6915 (19.2)	
Co-habitant with father-to-be			*p* < 0.0001		*p* < 0.0001		*p* < 0.0001
Yes	1,771,476 (94.9)	20,617 (91.8)		89,334 (83.4)		28,116 (79.4)	
No	95,654 (5.1)	1844 (8.2)		17,780 (16.6)		7293 (20.6)	
Missing	26,513	338		1873		676	
Education (years)			*p* < 0.0001		*p* < 0.0001		*p* < 0.0001
≤9	165,350 (9.4)	3020 (13.9)		33,420 (32.6)		13,877 (40.4)	
10–<12	194,730 (11.1)	3824 (17.7)		24,678 (24.0)		9991 (29.1)	
≥12	1,392,896 (79.5)	14,824 (68.4)		44,548 (43.4)		10,446 (30.4)	
Missing	140,667	1131		6341		1771	
Mother’s country of birth			*p* < 0.0001		*p* < 0.0001		*p* < 0.0001
Nordic	1,479,483 (78.2)	21,203 (93.1)		88,765 (81,5)		30,747 (85.3)	
Non-Nordic	412,381 (21.8)	1570 (6.9)		20,132 (18.5)		5314 (14.7)	
Missing	1779	26		90		24	
*Birth characteristics*							
Gestational age (weeks)			*p* < 0.0001		*p* < 0.0001		*p* < 0.0001
<37	102,326 (5.4)	1407 (6.2)		7744 (7.1)		3099 (8.6)	
37–<42	1,380,046 (72.9)	16,949 (74.3)		80,007 (73.4)		26,845 (74.4)	
≥42	411,271 (21.7)	4443 (19.5)		21,236 (19.5)		6141 (17.0)	
Birth weight (perc)			*p* < 0.0001		*p* < 0.0001		*p* < 0.0001
<10	125,656 (6.6)	1427 (6.3)		12,914 (11.9)		4958 (13.7)	
10–90	1,540,893 (81.4)	18,313 (80.3)		87,552 (80.3)		28,656 (79.4)	
>90	227,094 (12.0)	3059 (13.4)		8521 (7.8)		2471 (6.85)	

^a^Chi^2^ test was used to calculate *p* values for maternal and birth characteristics.

### Statistical analyses

The rates for post neonatal mortality and cause of death were calculated as the number of deaths divided by the number of infants. Chi^2^ test was used to calculate *p* values for maternal and birth characteristics. The logistic model was used to estimate crude and adjusted odd ratios (aORs) with 95% confidence interval (CI) for the association between tobacco use in early pregnancy and post neonatal mortality, SIDS, and SUID, as well as for the association between cessation of tobacco use before the antenatal booking and post neonatal mortality, SIDS, and SUID. Crude ORs were estimated on births with complete data on all covariates used in the first adjusted model, *n* = 1,883,726, (91% of the population). The first adjusted model included the covariates maternal age, parity, co-habitant with father-to-be, mother’s level of education at birth, and mother’s country of birth. We also wanted to investigate whether possible risks related to tobacco use were explained by preterm birth and SGA births. Therefore, in the second adjusted model, we additionally adjusted for gestational age and birth weight adjusted for gestational age. Further, we repeated our analyses with only term and non-SGA births included to study if potential associations were independent of effects on length of gestation or fetal growth.

To examine the potential effect of missing information on tobacco use, we also conducted analyses assuming the extreme cases that all missing values were either related to tobacco users or nonusers. All analyses were performed using SAS version 9.4 (Statistical Analysis Software version 9.4, SAS Institute, Inc, Cary, NC).

## Results

In the study, 145,072 (7%) of the mothers smoked and 22,799 (1.1%) used snuff in early pregnancy. More than 50% of smokers 3 months prior to pregnancy and almost 70% of previous snuff users had stopped smoking or using snuff at their first antenatal visit (data not shown). Maternal tobacco use, and especially heavy smoking, was more common among young mothers (≤20 years at delivery), mothers not co-habitant with the father-to-be, and mothers who were multiparous and had a low level of education (<12 years of schooling). Maternal smoking was more common among mothers born outside a Nordic country, whereas snuff use was more common among mothers born in a Nordic country. Preterm birth was more common among smoking and snuff using mothers, and SGA births were more common among mothers who smoked (Table 1).

The post neonatal mortality rate was 0.8 per 1000 live-born infants. The highest rates of post neonatal mortality, SIDS, and SUID were found among infants of mothers with low maternal age (<20 years at delivery), who were multiparous, not co-habitant with father-to-be, and with low level of education (<12 years of schooling). Further, post neonatal mortality, SIDS, and SUID were more common among preterm and SGA infants. Post neonatal mortality rates decreased with increasing gestational age. Of all the post neonatal deaths, almost 32% were preterm and 28% were SGA births (Table 2). Median age of post neonatal death was 3 months (interquartile range (IQR) 1–6). The median age of death due to SIDS was 2 months (IQR 1–3) and due to SUID 1 month (IQR 0–3) (Data not shown).

**Table 2:** Maternal and birth characteristics for post neonatal mortality, SIDS, and SUID, *N* = 2,061,154.

	Post neonatal mortality		SIDS		SUID	
	*N* = 1583, *n* (%)	Crude OR (95% CI)	*N* = 381, *n* (%)	Crude OR (95% CI)	*N* = 611, *n* (%)	Crude OR (95% CI)
*Maternal characteristics*						
Maternal age (years)						
<20	52 (0.18)	2.38 (1.72–3.30)	27 (0.09)	4.91 (3.08–7.83)	31 (0.11)	3.88 (2.54–5.90)
20–24	281 (0.11)	1.47 (1.26–1.72)	95 (0.04)	1.96 (1.47–2.60)	146 (0.06)	1.98 (1.58–2.50)
25–29	457 (0.07)	Reference	117 (0.02)	Reference	177 (0.03)	Reference
29–34	449 (0.06)	0.84 (0.73–0.96)	82 (0.01)	0.62 (0.46–0.83)	140 (0.02)	0.70 (0.55–0.88)
≥35	344 (0.08)	1.06 (0.91–1.23)	61 (0.01)	0.72 (0.52–1.00)	117 (0.03)	0.94 (0.74–1.20)
Parity						
1	567 (0.06)	0.81 (0.73–0.91)	117 (0.01)	0.69 (0.54–0.88)	215 (0.02)	0.84 (0.70–1.01)
2–3	809 (0.08)	Reference	193 (0.02)	Reference	301 (0.03)	Reference
≥4	207 (0.16)	2.07 (1.76–2.44)	71 (0.06)	3.17 (2.39–4.20)	95 (0.07)	2.77 (2.18–3.52)
Cohabitant with father-to-be						
Yes	1387 (0.07)	Reference	309 (0.02)	Reference	516 (0.03)	Reference
No	174 (0.14)	2.05 (1.74–2.43)	68 (0.06)	3.41 (2.58–4.51)	87 (0.07)	2.69 (2.12–3.42)
Missing	22	–	4			
Education (years)						
≤9	357 (0.17)	2.75 (2.42–3.13)	120 (0.06)	4.73 (3.73–5.99)	161 (0.07)	3.50 (2.88–4.24)
10–<12	232 (0.10)	1.66 (1.43–1.92)	60 (0.03)	2.18 (1.61–2.94)	93 (0.04)	1.88 (1.48–2.37)
≥12	873 (0.06)	Reference	172 (0.01)	Reference	313 (0.02)	Reference
Missing	121	–	29	–	44	
Mother’s country of birth						
Nordic	1124 (0.07)	Reference	329 (0.02)	Reference	495 (0.03)	Reference
Non-Nordic	457 (0.11)	1.44 (1.27–1.62)	51 (0.01)	0.51 (0.36–0.71)	113 (0.03)	0.84 (0.67–1.04)
Missing	2	–	1	–		
*Birth characteristics*						
Gestational age (weeks)						
<37	514 (0.46)	7.85 (6.99–8.82)	68 (0.06)	3.20 (2.41–4.26)	176 (0.15)	6.47 (5.34–7.83)
37–<42	875 (0.06)	Reference	163 (0.02)	Reference	356 (0.02)	Reference
≥42	194 (0.04)	0.76 (0.65–0.90)	50 (0.01)	0.66 (0.48–0.90)	79 (0.02)	0.74 (0.57–0.95)
Birth weight (perc)						
<10	451 (0.31)	5.32 (4.73–5.98)	68 (0.05)	2.66 (2.00–3.54)	126 (0.09)	3.38 (2.75–4.17)
10–90	1006 (0.06)	Reference	276 (0.02)	Reference	428 (0.03)	Reference
>90	126 (0.05)	0.84 (0.69–1.03)	37 (0.02)	0.85 (0.59–1.23)	57 (0.02)	0.89 (0.66–1.19)

Both maternal snuff use and smoking were associated with higher risk of post neonatal mortality. Snuff use in pregnancy was associated with 70% higher risk of post neonatal mortality in comparison to that of nonusers, aOR 1.71 (95% CI: 1.16–2.53). The highest risk of post neonatal mortality compared to that of nonusers was found among infants of heavy smokers, aOR 2.72 (95% CI: 2.17–3.40) (Table 3).

**Table 3:** Maternal tobacco use in early pregnancy and risk of post neonatal mortality, SIDS, and SUID, *N* = 2,061,514.

	*n* (%)	Crude^a^ OR (95% CI)	Adj. model 1^b^ OR (95% CI)	Adj. model 2^c^ OR (95% CI)
Post neonatal mortality^d^, *N* = 1583				
Nonuser	1274 (0.07)	Reference	Reference	Reference
Snuff user	27 (0.12)	1.83 (1.24–2.69)	1.71 (1.16–2.52)	1.65 (1.11–2.43)
Moderate smoker	185 (0.17)	2.54 (2.16–2.99)	1.89 (1.60–2.24)	1.60 (1.35–1.89)
Heavy smoker	97 (0.27)	4.06 (3.25–5.07)	2.72 (2.17–3.41)	2.15 (1.71–2.71)
SIDS, *N* = 381				
Nonuser	214 (0.01)	Reference	Reference	Reference
Snuff user	13 (0.06)	5.04 (2.81–9.03)	3.70 (2.06–6.65)	3.67 (2.04–6.59)
Moderate smoker	91 (0.08)	7.64 (5.93–9.89)	4.31 (3.26–5.70)	3.97 (2.99–5.26)
Heavy smoker	63 (0.17)	14.9 (11.1–20.1)	7.03 (5.04–9.79)	6.26 (4.48–8.75)
SUID, *N* = 611				
Nonuser	406 (0.02)	Reference	Reference	Reference
Snuff user	17 (0.07)	3.52 (2.14–5.81)	2.90 (1.75–4.80)	2.84 (1.72–4.71)
Moderate smoker	112 (0.10)	4.94 (3.98–6.13)	3.20 (2.53–4.05)	2.87 (2.26–3.63)
Heavy smoker	76 (0.21)	9.50 (7.34–12.3)	5.44 (4.10–7.22)	4.66 (3.50–6.19)

*CI* confidence interval, *OR* odds ratio.

^a^Crude odds ratios calculated with the same population as adjusted models.

^b^Adjusted for maternal age, parity, maternal education, cohabitant with father-to-be, mother’s country of birth.

^c^Adjusted for maternal age, parity, maternal education, cohabitant with father-to-be, mother’s country of birth, gestational age, birth weight according to gestational age.

^d^Population for post neonatal mortality was *n* = 2,058,647. Infants who died in the neonatal period *n* = 2867 were excluded.

**Fig. 2:**
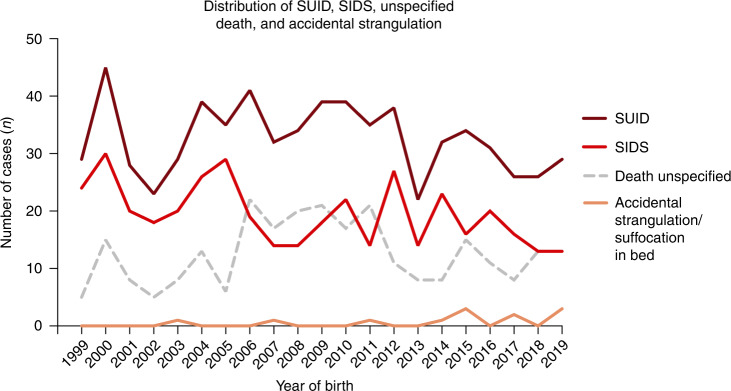
Distribution of cases of SUID, SIDS, accidental strangulation/ suffocation and unspecified death. Presentation of number of deaths/year in 1999–2019 of Sudden Unexpected Infant Death (SUID) as well as presentation of deaths of Sudden Infant Death Syndrome (SIDS), accidental strangulation/suffocation and unspecified death of the newborn.

There were a total of 611 cases of SUID. Of these, 60% were due to SIDS (*n* = 381), 11 cases of accidental strangulation in bed, and 219 death by unspecified cause. Frequency plot of SIDS, unspecified death, and accidental strangulation in bed by years is shown in Fig. 2. In Sweden, the frequency of reporting unspecified death or accidental strangulation cannot fully explain the fluctuations in SIDS cases. The total number of cases of SIDS and SUID has varied substantially over the years. There has been a gradual decrease, however, and since 2015, the number of SUID cases has been below 30 and SIDS cases below 20.

Snuff use in early pregnancy was associated with a more than tripled risk of SIDS, aOR 3.70 (95% CI: 2.06–6.65) compared to that of nonuse. Maternal smoking was also associated with higher risk of SIDS, aORs were 4.31 (95% CI: 3.26–5.70) and 7.03 (95% CI: 5.04–9.79) for moderate and heavy smoking, respectively. Maternal snuff use was associated with an almost tripled risk of SUID, aOR 2.90 (95% CI: 1.75–4.80). Maternal smoking was also associated with higher risk of SUID, with aORs 3.20 (95% CI: 2.53–4.05) and 5.44 (95% CI: 4.10–7.22) for moderate and heavy smoking, respectively. The associations were attenuated after adjustment for possible confounders in all analyses and were most pronounced in the analyses of maternal smoking (Table 3). The associations between maternal snuff use or smoking and post neonatal mortality, SIDS, and SUID in the subgroup analysis of only term and non-SGA infants (*n* = 1,824,357) were similar to the analyses of the full cohort (Table S1).

In infants of mothers who stopped using snuff or stopped smoking before the antenatal booking, there were no overall statistically significant higher risks of post neonatal mortality. However, among infants of mothers who had stopped using snuff before the antenatal booking the risk of SIDS was lower but still higher compared to that of nonusers, aOR 2.13 (95% CI: 1.04–4.34). In infants of mothers who had stopped smoking before the antenatal booking, the aOR was 1.35 (95% CI: 0.89–2.05) compared to that of nonusers. In infants of mothers who had stopped using snuff or smoking before the antenatal booking, risks of SUID was substantially lower, aOR 1.37 (95% CI: 0.73–2.58) and aOR 1.29 (95% CI: 0.95–1.74), respectively (Table 4). Results did not substantially differ for any of the outcomes regardless of whether individuals with missing information on tobacco use in pregnancy were assumed to be all tobacco users or all non-tobacco users (data not shown).

**Table 4:** Maternal tobacco use in early pregnancy and cessation of tobacco use before the antenatal booking and risk of post neonatal mortality, SUID, and SIDS, *N* = 1,996,980.

	*n* (%)	Crude^a^ OR (95% CI)	Adjusted^b^ OR (95% CI)	Adjusted^c^ OR (95% CI)
Post neonatal mortality^d^, *N* = 1514				
Nonuser	1072 (0.07)	Reference	Reference	Reference
Snuff user	22 (0.13)	1.95 (1.27–3.01)	1.81 (1.17–2.80)	1.75 (1.13–2.71)
Stopped using snuff	27 (0.07)	1.08 (0.73–1.59)	1.21 (0.82–1.79)	1.24 (0.84–1.84)
Smoker	263 (0.19)	2.94 (2.56–3.39)	2.09 (1.79–2.44)	1.73 (1.48–2.02)
Stopped smoking	130 (0.07)	1.13 (0.94–1.37)	1.03 (0.85–1.24)	1.02 (0.84–1.23)
SIDS, *N* = 354				
Nonuser	160 (0.01)	Reference	Reference	Reference
Snuff user	11 (0.06)	6.16 (3.25–11.7)	4.36 (2.29–8.31)	4.33 (2.27–8.26)
Stopped using snuff	8 (0.02)	2.20 (1.08–4.49)	2.13 (1.04–4.34)	2.15 (1.05–4.40)
Smoker	141 (0.10)	10.3 (8.16–13.1)	5.46 (4.15–7.18)	4.96 (3.77–6.54)
Stopped smoking	34 (0.02)	1.71 (1.14–2.56)	1.35 (0.89–2.05)	1.35 (0.89–2.04)
SUID, *N* = 579				
Nonuser	324 (0.02)	Reference	Reference	Reference
Snuff user	14 (0.08)	3.97 (2.28–6.91)	3.20 (1.83–5.60)	3.16 (1.81–5.53)
Stopped using snuff	10 (0.02)	1.36 (0.73–2.56)	1.37 (0.73–2.58)	1.40 (0.74–2.63)
Smoker	174 (0.13)	6.35 (5.24–7.70)	3.95 (3.17–4.91)	3.46 (2.78–4.32)
Stopped smoking	57 (0.03)	1.54 (1.14–2.07)	1.29 (0.95–1.74)	1.27 (0.93–1.72)

*CI* confidence interval, *OR* odds ratio.

^a^Crude odds ratios calculated with the same population as adjusted models.

^b^Adjusted for maternal age, parity, maternal education, cohabitant with father-to-be, mother’s country of birth.

^c^Adjusted for maternal age, parity, maternal education, cohabitant with father-to-be, mother’s country of birth, gestational age, birth weight according to gestational age.

^d^Population for neonatal mortality was *n* = 1,994,209. Infants who died in the neonatal period were excluded, *n* = 2771.

## Discussion

In this population-based cohort study, including >2 million infants, we found that maternal snuff use in pregnancy was associated with a >70% higher risk of post neonatal death and a tripled risk of SIDS compared to that of nonusers. The risks of snuff use and moderate smoking were of similar magnitude. The highest risks were found among infants of heavy smokers, with a more than sevenfold higher risk of SIDS compared to that of nonusers. Both maternal snuff use and smoking were associated with higher risk of SUID. In our population, the majority of SUID cases among tobacco users were due to SIDS. Cessation of smoking and snuff use before the antenatal booking was associated with lower risks of SIDS and SUID.

Despite a study population of more than two million infants, the number of SIDS cases was small. In the cohort, only 0.2 per 1000 live-born infants died of SIDS and <7% of women smoked and only 1% used snuff in pregnancy. However, despite small numbers, the estimates and confidence intervals are in agreement with international studies on smoking, post neonatal mortality, SIDS and SUID.^19–22^ Our results are in agreement with Anderson et al. who showed that infants of mothers who had stopped smoking in early pregnancy ran a reduced risk of SUID compared to those whose mothers continued to smoke.^20^ To speculate, the lower, but still elevated, risk observed in infants of mothers who had stopped using snuff before the antenatal booking may be due to residual confounding or environmental tobacco exposure. It is also likely that a proportion of women who used snuff or smoked pre-pregnancy and quit before pregnancy restarted later in pregnancy or in the postpartum period. It is also possible that among women who stopped using snuff or smoking as soon as they knew they were pregnant, the fetus had been exposed to snuff or cigarette smoking in very early pregnancy. As nicotine-acetylcholine receptors (nAChRs) are present as early as gestational week 4–5 in fetal development, before most women are aware of their pregnancy, nicotine may carry out teratogenic effects by binding these receptors, induce apoptosis, and affect cell programming.^23^

The associations between maternal snuff use and SIDS remained fairly stable after adjusting for possible confounders, whereas the associations with especially heavy maternal smoking were considerably attenuated in the adjusted analyses. Snuff use is not to the same extent as smoking associated with low socioeconomic status.^24^ Further, snuff use in pregnancy is associated with increased risk of preterm birth, but only with modest effects on birth weight,^25^ which may explain why the risk of SIDS associated with snuff use was only slightly attenuated when adjusting for preterm and SGA births. By stratifying for gestational age and birth weight, we may have introduced collider stratification bias, suggesting those results must be interpreted with caution. However, the findings were similar to those of the full cohort, indicating that preterm birth and birth weight may not fully explain the association between nicotine use and SIDS.

The risk of SIDS is multifactorial, and prone sleeping position, bed sharing, and prenatal and postnatal exposure to tobacco smoke are regarded as important risk factors.^1,26^ Most SIDS deaths occur at around 2–4 months of age when the brain and cardiorespiratory systems mature.^27^ It has been suggested that SIDS is related to disturbed autonomous control and disturbances in the serotonergic neural development.^1^ Maternal smoking has been associated with similar disturbances in the cardiorespiratory and serotonergic system.^1,28^ Further, animal studies of prenatal nicotine suggest that nicotine is involved in the mechanisms behind smoking-related risks of SIDS.^5,23^ Maternal snuff use has been associated with neonatal apnea and disturbed heart rate variability.^12,15^ The common substance in cigarette smoke and snuff is nicotine. The peak plasma concentration between cigarettes and snuff is similar. However, whereas smoking generates short peaks (30 min) of high plasma concentration intermittently during the day, snuff has a slower release and a longer peak duration, 1.5–2 h.^29^ Snuff use leads to higher accumulated nicotine levels and a more continuous exposure on the nicotine receptors, compared with short, intermittent exposure in smoking, which may explain differences between effects of smoking and snuff use.^29,30^ Our findings support the hypothesis that nicotine is involved in the mechanisms behind the increased risk of post neonatal mortality and SIDS in infants of smokers.

### Strengths and limitations

The major strength with our study is the large study population of two million births and the nationwide design. Further, the prospectively collected tobacco information in early pregnancy precludes recall bias. This is, to our knowledge, the first study exploring snuff use in pregnancy and risk of post neonatal mortality, SIDS, and SUID. By linking several Swedish national registers, we were able to control for several important confounders, including socioeconomic status, parity, and maternal age.

Nicotine replacement therapy (NRT) is often regarded a safe alternative in pregnancy. However, safety studies of NRT use in pregnancy have not been conclusive due to lack of compliance to NRT. Cessation of smoking has not proved successful at used doses of NRT in randomized studies. The Cochrane systematic review by Clair et al.^31^ concluded that studies with higher doses of nicotine were needed to study safety. The metabolism of nicotine is elevated in pregnancy, which may partly explain why smoke cessation in pregnancy is difficult to obtain with NRT.^32^ The widespread use of snuff in Sweden, also in pregnancy, provides a unique opportunity to specifically study the effect of nicotine separated from the effect from other combustion products in smoking.

There are also some limitations of the study. First, the diagnoses were collected from the Cause of Death Register, without further validation through the medical records. However, all infant deaths are thoroughly investigated in Sweden and the Cause of Death Register has been validated.^18^ Nevertheless, crime scene investigations are not routinely performed in Sweden following SIDS/SUID^17,33^ and potentially cases of accidental and strangulation in bed could have been misclassified as SIDS in the current study.^33^ Second, the self-reported tobacco information was not validated by cotinine levels. However, self-reported smoking and snuff use in pregnancy has been proved a valid proxy for nicotine levels in blood.^34,35^ In addition, we lacked information on the number of snuff pouches consumed daily. However, a previous validation study, based on a sub-cohort of the women included in the MBR register, showed that the mean nicotine dose of women who continued to use snuff during pregnancy was 46 mg/day in early pregnancy (corresponds to 5–6 pouches/daily) and 36 mg/day (corresponds to 3–4 pouches daily) among those who quit in early pregnancy.^35^ The validation study also showed that women using NRT in pregnancy continued to consume snuff. Among the women who stopped using snuff, none was using NRT.^35^ This is in agreement with other studies on NRT use in pregnancy and smoke cessation lacking strong evidence for successful smoke cessation with NRT use in pregnancy.^31,36^ Third, we had information on tobacco use 3 months before pregnancy and at the antenatal booking, but we lack information on when in that time span the women stopped their tobacco use. Finally, we cannot make definitive causal conclusions, given the lack of information on important confounding factors, including alcohol and substance use, and prenatal and postnatal exposure to environmental tobacco smoke. Missing mandatory education, as well as being a teenage mother, was highly associated with both tobacco use in pregnancy, especially heavy smoking, and risk of postnatal death and SIDS. It is not possible to disentangle the risk of smoking in pregnancy from other possible neonatal health hazards in this group of women. Sibling comparisons or other family design analyses may have helped account for bias due to unmeasured and residual confounding shared by siblings but were not possible to conduct due to limited power.^37^

The adverse effects of nicotine on pregnancy and the developing fetus are often neglected in discussions of smokeless tobacco or ENDS as means of smoke cessation and harm reduction.^38^ In the US, e-cigarettes are now more popular than conventional cigarettes among young adults.^7^ With growing knowledge about the detrimental effects of non-combustible nicotine in the form of snuff, caution should be recommended regarding use of other sources of nicotine in pregnancy as well. No safe level of prenatal nicotine has been established. The use of new tobacco products and other nicotine sources place high demands on society and health care professionals regarding information and preventive strategies.

## Conclusion

Snuff use and smoking in pregnancy were associated with increased risks of post neonatal mortality, SIDS, and SUID. The risks of snuff use and moderate smoking were of similar magnitude. Cessation of smoking and snuff use before the antenatal booking was associated with lower risks of SIDS and SUID. Our findings support the hypothesis that nicotine contributes to elevated risk of SIDS. Prevention strategies should focus on all forms of nicotine use in pregnancy. Nicotine in all forms should be avoided in pregnancy.

## Supplementary information

Supplemental table 1

## Data Availability

Restrictions apply to the availability of these data. Data were obtained from the Swedish National Board of Health and Welfare and are available from the authors with the permission of the Swedish National Board of Health and Welfare.
