# Supplementary material for: Association of maternal snuff use and smoking with Sudden Infant Death Syndrome: a national register study
Source: Pediatr Res. 2023 Feb 9;94(2):811–9. doi: 10.1038/s41390-022-02463-4 (PMC10382311; doi:10.1038/s41390-022-02463-4)
Supplement: Supplementary file 1 — Supplemental table 1 [file 41390_2022_2463_MOESM1_ESM.docx]

| **Supplemental table 1.** Maternal tobacco use and post neonatal mortality, SIDS and SUID in term, non-SGA infants, N= 1,824,357 | | | | | | | |
| --- | --- | --- | --- | --- | --- | --- | --- |
|  |  | **n(%)** |  | **Crude**^a^  **OR 95% CI** |  | **Adj. model 1**^b^  **OR 95%CI** |  |
| **Post neonatal mortality**^c^**, N=837** |  |  |  |  |  |  |  |
| Nonuser |  | 680(0.04) |  | reference |  | reference |  |
| Snuff user |  | 21(0.10) |  | 2.66(1.70-4.15) |  | 2.39(1.53-3.73) |  |
| Moderate smoker |  | 82(0.09) |  | 2.35(1.85-2.97) |  | 1.68(1.32-2.14) |  |
| Heavy smoker |  | 54(0.19) |  | 4.58(3.39-6.20) |  | 2.90(2.13-3.95) |  |
| **SIDS, N=264** |  |  |  |  |  |  |  |
| Nonuser |  | 165(0.01) |  | reference |  | reference |  |
| Snuff user |  | 10(0.05) |  | 4.79(.244-9.38) |  | 3.50(1.79-6.83) |  |
| Moderate smoker |  | 51(0.06) |  | 6.02(4.38-8.29) |  | 3.35(2.38-4.72) |  |
| Heavy smoker |  | 38(0.13) |  | 12.7(8.70-18.6) |  | 5.94(3.88-9.10) |  |
| **SUID, N=369** |  |  |  |  |  |  |  |
| Nonuser |  | 250(0.01) |  | reference |  | reference |  |
| Snuff user |  | 12(0.06) |  | 3.95(2.15-7.23) |  | 3.15(1.73-5.75) |  |
| Moderate smoker |  | 61(0.07) |  | 4.79(3.60-6.38) |  | 3.02(2.23-4.10) |  |
| Heavy smoker |  | 46(0.16) |  | 10.3(7.37-14.5) |  | 5.67(3.90-8.23) |  |
| Note: CI, confidence interval; OR, odds ratio  ^a^Crude odds ratios calculated with the same population as adjusted models.  ^b^Adjusted for maternal age, parity, maternal education, cohabitant with father-to-be, mother’s country of birth  ^c d^Population for neonatal mortality was n=1,823,555. Infants who died in the neonatal period were excluded, n=802. | | | | | | | |
